# Sero-prevalence of yellow fever and related Flavi viruses in Ethiopia: a public health perspective

**DOI:** 10.1186/s12889-018-5726-9

**Published:** 2018-08-14

**Authors:** Mesfin Mengesha Tsegaye, Berhane Beyene, Workenesh Ayele, Almaz Abebe, Israel Tareke, Amadou Sall, Sergio Yactayo, Messeret E. Shibeshi, Erin Staples, Desalegn Belay, Abrham Lilay, Abebe Alemu, Emana Alemu, Adugna Kume, Alemnesh H/Mariam, Olivier Ronveaux, Mesfin Tefera, Woubayehu Kassa, Abyot Bekele Weyessa, Daddi Jima, Amha Kebede, Adamu Tayachew

**Affiliations:** 1 Ethiopian Public Health Institute, Addis Ababa, Ethiopia; 2 World Health Organizations, Country Office, Addis Ababa, Ethiopia; 3 Institute Pasteur of Dakar, Dakar, Senegal; 4 World Health Organizations, Geneva, HQ Switzerland; 5 World Health Organizations, Zimbabwe, IST, Harare, Zimbabwe; 6 Centers for Disease Control and Prevention, Atlanta, USA; 7 IST/AFRO, Ouagadougou, Burkina Faso

**Keywords:** Yellow fever, Flaviviruses, Risk assessment, Ethiopia

## Abstract

**Background:**

Yellow fever (YF) is a viral hemorrhagic fever, endemic in the tropical forests of Africa and Central and South America. The disease is transmitted by mosquitoes infected with the yellow fever virus (YFV). Ethiopia was affected by the largest YF outbreak since the vaccination era during 1960–1962. The recent YF outbreak occurred in 2013 in Southern part of the country. The current survey of was carried out to determine the YF seroprevalence so as to make recommendations from YF prevention and control in Ethiopia.

**Methodology:**

A multistage cluster design was utilized. Consequently, the country was divided into 5 ecological zones and two sampling towns were picked per zone randomly. A total of 1643 serum samples were collected from human participants. The serum samples were tested for IgG antibody against YFV using ELISA. Any serum sample testing positive by ELISA was confirmed by plaque reduction neutralization test (PRNT). In addition, differential testing was performed for other flaviviruses, namely dengue, Zika and West Nile viruses.

**Result:**

Of the total samples tested, 10 (0.61%) were confirmed to be IgG positive against YFV and confirmed with PRNT. Nine (0.5%) samples were antibody positive for dengue virus, 15(0.9%) forWest Nile virus and 7 (0.4%) for Zika virus by PRNT. Three out of the five ecological zones namely zones 1, 3 and 5 showed low levels (< 2%) of IgG positivity against YFV. A total of 41(2.5%) cases were confirmed to be positive for one of flaviviruses tested.

**Conclusion:**

Based on the seroprevalence data, the level of YFV activity and the risk of a YF epidemic in Ethiopia are low. However additional factors that could impact the likelihood of such an epidemic occurring should be considered before making final recommendations for YF prevention and control in Ethiopia. Based on the results of the serosurvey and other YF epidemic risk factors considered, a preventive mass vaccination campaign is not recommended, however the introduction of YF vaccine in routine EPI is proposed nationwide, along with strong laboratory based YF surveillance.

## Background

Yellow fever (YF) is a viral hemorrhagic fever, endemic in the tropical forests of Africa and Central and South America [1]. Mosquitoes are known vectors of yellow fever virus. The clinical manifestations of YF include fever, headache, jaundice, muscle pain, nausea, vomiting and fatigue. A small proportion of patients who contract the virus develop severe symptoms and approximately half of those die within 7 to 10 days [2]. The precise extent of illness and death attributable to YF is unknown. Estimates by World Health Organization (WHO) and other international agencies suggest that only 1–2% of cases are reported [3]. Unfortunately, an YF outbreak may not be detected because the clinical manifestation overlaps with the symptoms of other hemorrhagic fever, viral hepatitis, malaria, and leptospirosis, and this leads to misdiagnosis. In another scenario, yellow fever mild cases may also remain undetected because the patients are often treated at home and do not seek care in a health facility. In general it is estimated that there are 200,000 YF cases annually and that 30, 000 deaths occur each year in 44 countries, almost all of them occur in sub-Saharan Africa [4]. A modeling study based on African data sources estimated the burden of YF during 2013 was 84,000–170,000 severe cases and 29,000–60,000 deaths [5].

Large epidemics of YF occur when infected people introduce the virus into heavily populated areas with high mosquito density, where due to lack of vaccination, most people have little or no immunity [2]. In Ethiopia, the first confirmed YF outbreak occurred in 1959 in Wollega province (currently Oromiya Regional State) which was due to an extension of an outbreak in the Blue Nile country of Sudan in the same year [6]. A sylvatic epidemic ensued between 1960 and 62 in almost all the rural districts of the then Gamu-Goffa province,Shoa (Wallamo District), Kaffa (Coulo-Conta District) and Wollega (Didessa Region) [6]. The outbreak began at the Sudanese border in the Omo and Didessa river valleys and was transmitted by the vectors *A*. *simpsoni* and *A. africanus* [7]. The spread of the epidemic was possibly linked to movements of monkeys, including baboons, leaving the forests and plantations in search of food. This was the largest reported YF epidemic ever recorded globally since the beginning of the vaccination era [7]. The outbreak affected 10% of the 1,000,000 residents of South-Western Ethiopia, a population without any background immunity and had an estimated case-fatality rate (CFR) of 30% [6]. In 1966, YF outbreak was reported in Arba-Minch district, east of the previous outbreak, in which a total of 2200 human cases were reported with about 20.5% deaths [8]. It was hypothesized that baboons from the previous outbreak area imported the virus to the district and the virus persisted in the area from1959–1966 [8]. Approximately 10 years after the 1960–66 outbreaks, Wood and Lee visited the outbreak sites and found specific neutralizing antibodies in 22% of unvaccinated people including from a child born after the outbreak, as well as in baboons and Colobus monkeys [9], suggesting persistence of silent YFV transmission.

In 2013, nearly half a century following the historic YF outbreak in Ethiopia, there was another confirmed YF outbreak in South Omo Zone of Southern Nations, Nationalities and Peoples Regional State (SNNPR). In this most recent outbreak there were a total of 141 YF suspected cases of which 7 were subsequently laboratory confirmed, with 43 deaths reported that was attributed to YFV infection [10]. The affected area in the recent YF outbreak corresponds to part of the same area which had been affected by the previous large YF outbreak in the country nearly fifty years ago. What the recent YF outbreak in South Omo, Ethiopia, has shown is that despite the long years of silence, YFV has been circulating in the area throughout this time, with the potential to cause outbreaks at any time. The current study was carried out to determine the sero prevalence of YF and related flavi viruses based on recent exposure to YFV and the flavi viruses,understand the risk of YF epidemics and based on the findings recommend YF control strategy in Ethiopia,

## Methodology

As a methodological approach, the WHO protocol for Risk assessment on Yellow Fever Virus circulation in endemic countries was followed [11]. Data and information on YFV activity were obtained by surveying humans and the information was aggregated according to distinct ecological zones.

### Ecological zone demarcation and selection of study sites

**Fig. 1:**
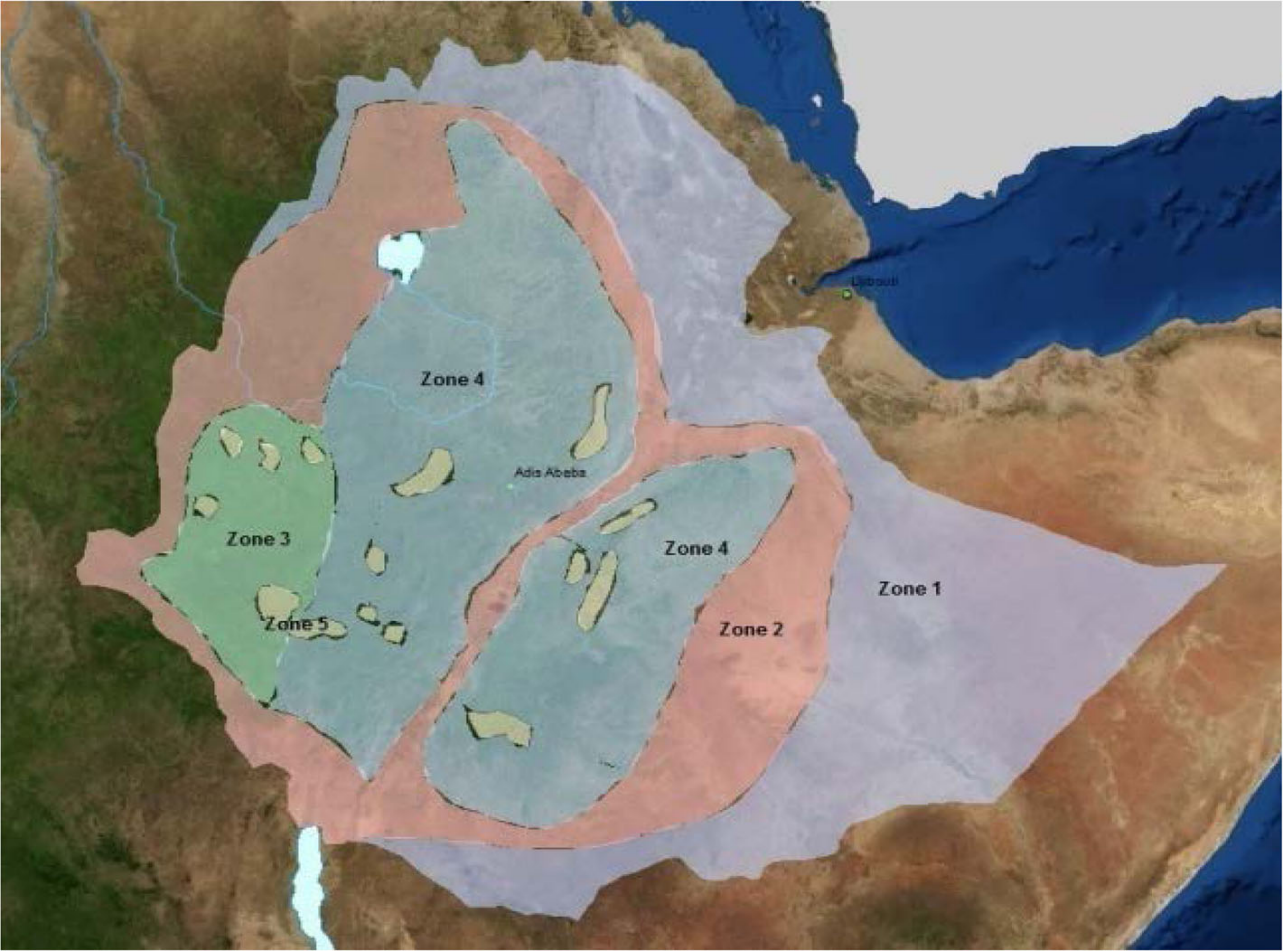
Map of Ethiopia Showing Demarcated Ecological Zones

A multistage cluster design was utilized and the initial stage involved identifying distinct ecologic zones within the country based on rainfall, vegetation cover and elevation. Consequently, the country has been divided into 5 distinct Ecological Zones based on the above criteria (Fig. 1).

**Fig. 2:**
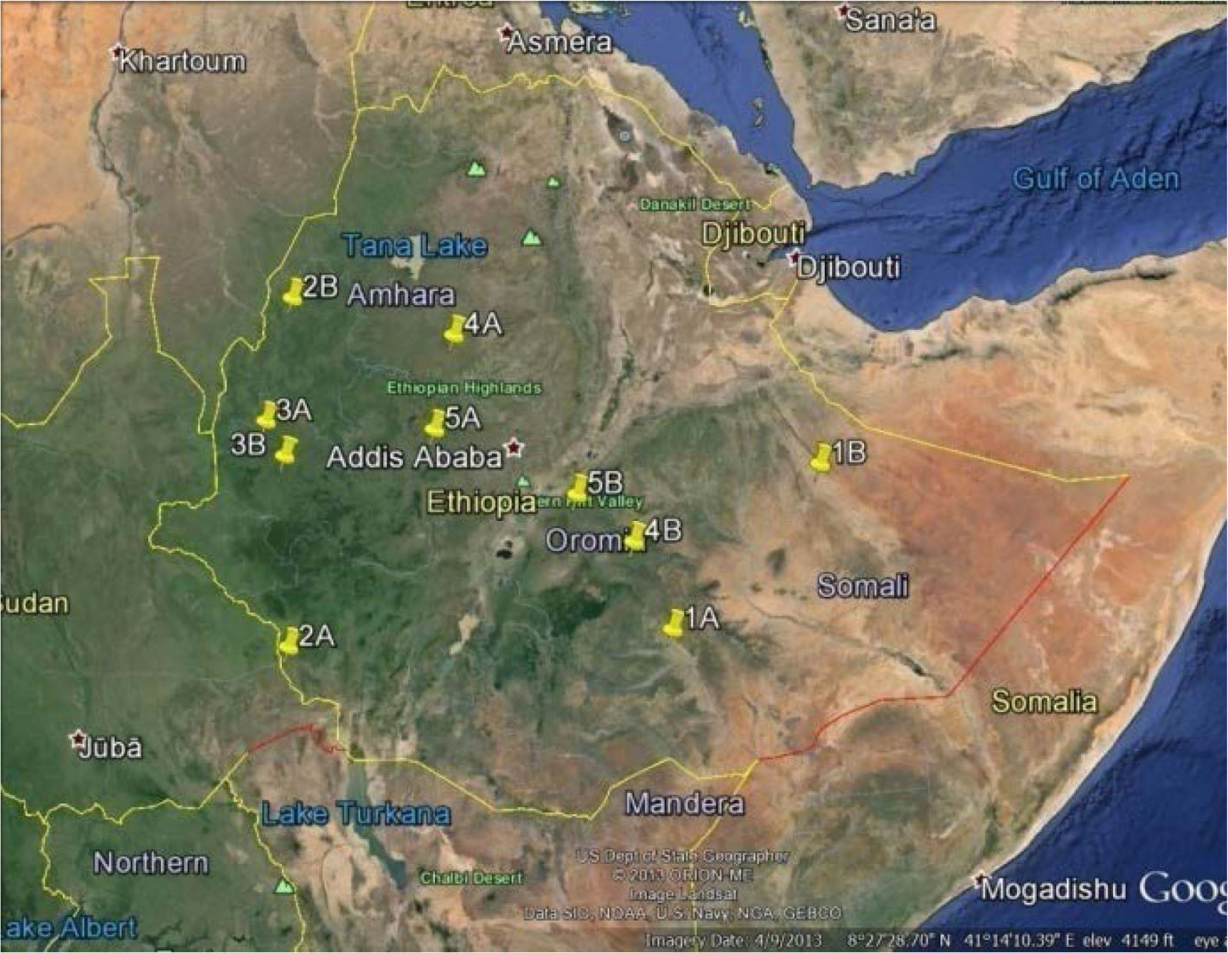
Map of Ethiopia showing study sites selected in each Ecological Zone

The next step of the site selection process involved using a random point generator in ArcGIS to randomly pick two locations per Ecological Zone for sampling. Two sampling points were picked per Zone (Fig. 2) to increase the likelihood of the representativeness of the data for the Ecological Zone and to ensure that field teams would be able to access the sites and perform the assessment within a reasonable time frame. If the random point was in an area where there was a recent vaccination campaign conducted, another random point was generated for the Zone (Table 1).

**Table 1:** Study Sites Selected by Ecologic Zone

Ecological Zone	Random points in Ecological Zones	GPS coordinates	Nearest town/city selected	Regional State
1	1A	42°51′6.0178″E, 5°57′50.0569”N	Gode	Somali Region
1	1B	43°11′5.732″E,8°16′10.5221”N	Degehabur	Somali Region
2	2A	35°2′44.4465″E,5°57′50.0569”N	MizanTeferi	SNNPR
2	2B	35°21′40.3682″E,11°13′0.1394”N	Pawe	BenishangulGumuz
3	3A	34°52′46.5932″E,9°22′44.731”N	Mendi	Oromia
3	3B	35°9′12.2294″E,8°50′30.0361”N	Dembidollo	Oromia
4	4A	37°50′ 34.7642″E,0°30′21.3493”N	Debremarkos	Amhara
4	4B	40°21′45.1564″E,7°17′55.5894”N	Robe (Bale Zone)	Oromia
5	5A	37°28′45.706″E,9°7′8.5883”N	Guder	Oromia
5	5B	39°33′37.5173″E,8°2′39.1985”N	Robe (Arsi Zone)	Oromia

### Sample size estimation

For the human sero-surveys, sample size was calculated per Zone based on the population size and estimated seroprevalence based on previous studies of prevalence of YF antibodies in the human population that specific study Zone. Based on data available for the population distribution, it was estimated that each Zone would have at least 100,000 persons. To compensate for households not being available for sampling and for individual refusal, 15% over sampling was calculated. In the sample size calculation, a design effect of 2 was used to account for clustering. After the sample size was calculated for each Ecological Zone, the number was divided between the two study sites selected per Zone and then stratified based on the population estimates of the cities and associated villages within each of the study sites.. The estimated seroprevalence and 95% CI for each zone was determined based on 1) the history of known outbreaks of YF disease [6–8], 2) information from previous serosurveys [9], and 3) data on ecology and elevation of the Ecological Zones [6–10]. Due to the lack of existing data for Zones 3 and 5, the seroprevalence was estimated based on the ecology and elevation and they were judged to have a low seroprevalence (Table 2).

**Table 2:** Sample Size Calculations per Zone

Zone/study site	Estimated Seroprevalence (95% CI)	Estimated sample size	15% oversampling number	Number of samples per Zone	Number of samples per Site
Zone 1	10% (3–17%)	142	21	164	
A Gode					82
B Degehabur					82
Zone 2	20% (5–35%)	54	8	62	
A MizanTeferi					31
B Pawe					31
Zone 3	3% (1–5%)	558	84	642	
A Mendi					321
B Dembidollo					321
Zone 4	12% (5–19%)	166	25	192	
A Debremarkos					96
B Robe (Bale Goba)					96
Zone 5	3% (1–5%)	558	84	642	
A Guder					321
B Robe (Arsi Zone)					321

### Data and sample collection

Multidisciplinary teams consisting of epidemiologists, virologists/laboratory personnel from the Ethiopian Public Health Institute (EPHI), and phlebotomists from health facilities in the study sites were trained on data collection tools and were involved in the data collection and sampling of humans in the study locations from May to July 2014.

### Household selection and human sampling

On arrival at a study town/city, a nearby village associated with the town was randomly selected based on its proximity to the town. At the same time the average household size in the area was also assessed. The number of households to visit for sampling in the study site was determined based on the pre-calculated sample size for the site and on the average size of households in the study area. The number of households to visit for sampling in the town and village was stratified by the population size of the town and the village. A random number generator was used to select specific households to sample. In some cases in order to ensure adequate power, the random number generator was used to add more households to the number initially calculated for visiting.

Blood sample was collected from all individuals greater than or equals to 5 years of age in the selected households after they were requested to give their consent to provide a blood sample to measure neutralizing antibodies against YFV. In addition, basic demographic information and history of YF vaccination or disease was also collected from each sampled individual. Five to ten milliliters (ml) of blood were collected from adults and children > 10 years and 1–3 ml were collected from children less than 10 years but above 5 years. All specimens were collected by a trained phlebotomist using a standard sterile technique. The blood samples were collected in serum separator tubes. The serum was separated and transferred to an appropriately labelled cryovial while the clotted blood component was appropriately disposed off as biohazard waste. The serum was stored in a freezer in the field immediately after collection and transported to the central laboratory at EPHI maintaining the cold chain throughout.

### Study inclusion and exclusion criteria

All person ≥5 years of age living in the study site were eligible for inclusion. Children aged < 5 years and those who were approached about the study but did not provide their consent to have their blood sample drawn were excluded.

### Serum testing

All serum specimens were shipped to the WHO reference laboratory for Arboviruses and Viral Hemorrhagic Fever Viruses at Institute Pasteur in Dakar, Senegal for laboratory analysis. The samples were tested for YFV-specific IgG antibodies using enzyme-linked immunoassay (ELISA). All samples testing positive for YFVIgG were then analysed for neutralizing antibody to YFV by the Plaque Reduction Neutralization Test (PRNT).

Given the potential for cross-reactive antibodies within the flaviviral genus, any sample testing positive for YFV-IgG was assessed for antibodies against other flaviviruses, namely dengue virus (DENV), West Nile virus (WNV) and Zika virus (ZIKAV) by ELISA. When any of these tests were positive, then the neutralizing antibody against the virus was assessed by PRNT for confirmation.

### Data analysis

Data was analysed by frequency distribution (frequency counts and percentages). Statistical analysis was performed using Microsoft offices excel. The frequency of YFV-specific antibodies was assessed by participant, Ecological Zone and demographics, including age and sex. Persons reporting a history of YF vaccination were excluded from the calculations of the proportion of the population who had YFV antibodies due to natural infection. If a person had neutralizing antibodies against multiple flaviviruses, IgG positivity (confirmed by PRNT), and differences in the antibody titres were used to determine the most likely infecting agent.

## Results

### Serosurvey results

A total of 1645 individuals from 426 households, living in the 10 study sites of the 5 Ecological Zones, and who consented to participate in the study were sampled. Two participants, both from Zone 4A (DebreMarkos town), had a YF vaccination history and therefore were excluded. Table 3 shows the gender and age distribution of the study participants by Ecological Zone where a total of 1048 (64%) females and 595 (36%) males participated. In all of the study Zones, most participants were in the 15–39 years age category which was 51% of the study participants, followed by the younger age group 6–14 years, which represented 30% of the participants.

**Table 3:** Gender and Age Distribution of Study Participants by Ecological Zone

Ecological Zone	1	2	3	4	5	Total
Sex	N (%)	N (%)	N (%)	N (%)	N (%)	N (%)
Male	62(41)	23(36)	238(36)	42(36)	231(35)	596(36)
Female	90(59)	41(64)	418(64)	74(64)	426 (65)	1049(64)
Total	152	64	656	116	657	1645
Age Group						
5–14	46(30)	21(34)	191(29)	39(34)	197(30)	494 (30)
15–39	67(45)	35 (55)	337(51)	49(42)	342(52)	830(51)
40–64	31(20)	7(11)	103(16)	26(22.4)	107(16)	274(17)
≥ 65	8(5)	1(1.5)	25(3.8)	2(1.7)	11(1.7)	47(2.8)
Total	152	64	656	116	657	1645

**Fig. 3:**
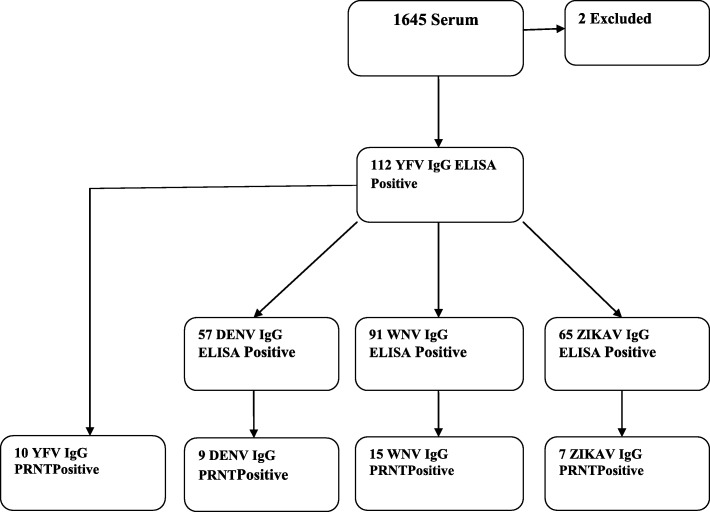
Results of Laboratory Investigation of the Serum Samples

Following the screening for the serum samples by ELISA, One hundred twelve samples were positive for IgG against YFV. The results of ELISA test for the other flavi viruses as well as findings of the confirmatory PRNT are also shown in Fig. 3: Results of Laboratory Investigation of the Serum Samples.

Table 4 shows the results of the Yellow Fever IgG antibody test by Ecological Zone. Ecological Zones 1, 3 and 5 showed low levels of IgG positivity against YFV (< 2%) while the other two Zones showed no evidence of viruses circulation.

**Table 4:** YF IgG Positivity by Ecological Zone

Ecological Zone	No. of Samples Collected	Number of Positives	Study sites in Zones (No. of Positives)	Positivity Rate (%)
1	152	2	1A(1), 1B [1]	1.3
2	64	0	2A(0),2B (0)	0.0
3	656	7	3A [4], 3B [3]	1.1
4	114	0	4A (0), 4B (0)	0.0
5	657	1	5A [1],5B (0)	0.2
Total	1643	10		0.6

Of the total samples tested, 10 (0.6%) were confirmed to be IgG positive against YFV, 9 (0.5%) against Dengue Virus (DENV), 15(0.9%) against West Nile Virus (WNV) and 7(0.4%) against Zika Virus (ZIKAV) by PRNT. A total of 41(2.5%) cases were confirmed to be positive for one the flaviviruses tested (Table 5).

**Table 5:** Proportion of Participants with Confirmed Flaviviruses Infections by Ecologic Zone

Ecological zone	Zone 1 *N* = 152	Zone 2 *N* = 64	Zone 3 *N* = 656	Zone 4 *N* = 116	Zone 5 *N* = 657	Total *N* = 1645
Flavivirus confirmed antibodies	n (%)	n (%)	n (%)	n (%)	n (%)	n (%)
YFV	2 (1.3)	0(0)	7 (1.1)	0(0)	1 (0.2)	10 (0.6)
DENV	8 (5.3)	0(0)	1 (0.2)	0 (0)	0 (0)	9 (0.5)
WNV	3 (2.0)	1 (1.6)	11 (1.7)	0 (0)	0 (0)	15 (0.9)
ZIKAV	0(0)	1 (1.6)	5 (0.8)	0(0)	1 (0.2)	7 (0.4)
Total	13 (8.6)	2 (3.1)	24 (3.7)	0 (0)	2 (0.3)	41 (2.5)

Among the 10 YF antibody positives, all age groups were affected. YFVseropositivity was highest (0.2%) for the 40–64 years age category and in terms of gender, 6 and 4 were seropositive from female and male participants, respectively (Table 6).

**Table 6:** Yellow Fever Virus Seropositivity by Sex and Age Group

Ecological Zone	1 N = 152	2 N = 64	3 N = 656	4 N = 116	5 N = 657	Total N = 1645
Sex	n (%)	n (%)	n (%)	n (%)	n (%)	n (%)
Male	0 (0)	0 (0)	3(0.5)	0 (0)	1(0.2)	4(0.2)
Female	2 (1.3)	0 (0)	4(0.6)	0 (0)	0 (0)	6(0.4)
Age Group						
6–14	0(0)	0(0)	0(0)	0(0)	1 (0.2)	1(0.1)
15–39	1(0.1)	0(0)	1(0.2)	0(0)	0(0)	2(0.1)
40–64	1(0.1)	0(0)	3(0.5)	0(0)	0(0)	4(0.2)
≥ 65	0(0)	0(0)	3(0.5)	0(0)	0(0)	3(0.2)

## Discussion

This serosurvey has provided the platform for understanding the seroprevalence of YF and related flaviviruses in the country, namely DENV, WNV and ZIKAV based on recent or previous exposure to the viruses. When we see the study participants which were positive for IgG against YFV, the smallest age was 11 year male (data not shown here), implying that YFV has been circulating just at least as recently as a decade ago and there is a high probability that the virus is there circulating, or hiding in the reservoir host silently, with the potential to cause an epidemic. The same is true for the related flavi viruses considered in this study too, for example the smallest age with IgG positivity for Zika virus was a nine year old girl (data not shown here), which also shows recent circulation of the viruse, with the probability of current circulation.

Three Ecological Zones namely, 1, 3 and 5 showed low levels of IgG positivity (< 2%). By contrast no YFV seropositivity was detected in the mostly contiguous Ecological Zones in the center of the country, namely Zones 2 and 4. One of the Ecological Zones with YFV seropositivity was Ecological Zone 1 and Degehabur town (1B) was one of the study sites sampled within the Zone. Historically, a suspected YF epidemic causing a severe and fatal illness with jaundice occurred in Degehabur in 1943 without clinical observation by competent observers or laboratory tests [6]. A serological survey carried out after the 1943 suspected YF epidemic detected YFV neutralizing antibodies in 4 out of 29 (13.8%) adults in that same year and in 3 out of 22 (14%) individuals from the same locality in 1944 [12, 13]. The prevalence of YFV antibodies found in Degehabur town (1.3%) was markedly lower than the prevalence from the 1943 study (14%), indicating that there has been a sharp decline in YFV seroprevalence over time in this region. This decline in prevalence can also be explained by the advances in laboratory test methods used over the years to detect and confirm antibodies against YFV.

Ethiopia has been affected by historic YF outbreaks that occurred in the Western and South-Western areas of the country between 1959 and 1966, and more recently in 2013 in South Omo Zone, an area that geographically corresponds to the historic outbreak region. In the Ecological Zone classification used in this sero prevalence study, Ecological Zones 2, 3, 4 and 5 share some geographical areas with the part of the country which has been the epidemic foci of the previous historic YF outbreaks. In the current study, these ecological zones either did not show any YFV seropositivity (Ecological Zones 2 and 4) or exhibited only a low YF seroprevalence (Ecological Zones 3 and 5). Approximately 10 years after the 1960–66 outbreaks, Wood and Lee [9] visited the historic YF outbreak sites and found specific neutralizing antibodies in 22% of unvaccinated people, including in a child born after the outbreak, again highlighting the fact there has been a decline in YF seroprevalence over time. However it should be noted that the difference in seroprevalence rates between the current study and previous ones may partly be related to differences in methods of laboratory analysis used and also the stages of the epidemic in the previous outbreaks. In a recent YF risk assessment done in two provinces in Zambia found YFV seroprevalence of 0.3%, which is also considered a low prevalence and was 96 to 98% lower seroprevalence compared to previous studies done in a similar provinces [14], consistent with our finding. But a similar risk assessment undertaken in Central African Republic (CAR) found a 13.3% seroprevalence of antibodies against YFV (employing a confirmatory PRNT test) in unvaccinated study participants [15] and therefore compared to the finding in CAR, Ethiopia has lower prevalence of YFV circulation based on our current survey. In another arbovirus seroprevalence study in Kenya by Luke et al. [16] in 2011 found 9.2% were YFV IgG positive, which is higher than the prevalence rate reported in our study, but one of the limitations of the Kenyan study was not having employed the PRNT confirmatory test to report the findings. Thus it is likely that the true YFV sero-prevalence could have been lower. The current result of not finding any virus activity in some Ecological Zones or else, finding only low YFV activity in others has to be interpreted with caution. In East Africa, YF remains characterized by unpredictable focal periodicity and a precarious potential for large epidemics [17]. Thus the absence of any virus activity, or its relatively low presence, belies any threat of an outbreak. Of course, the fact that our results showed presence of YFV circulation in 3 Ecological zones, albeit at a low prevalence, but none in 2 Zones, does not preclude the possibility of any of these zones experiencing YF outbreaks.

In addition to determining the YFV seroprevalence, this study has also been used to determine the previous exposure to other closely related flaviviruses which have a high degree of cross-reactivity with YFV. At a national level, this study reports for the first time the prevalence of other flaviviruses. Ecological Zone 4, in addition to being free of YFV antibodies, showed no positivity for any of the other flaviviruses, while in Ecological Zone 3 there was circulation of all the flaviviruses at a relatively low level. The positivity for all the viruses appeared to be low in all Ecological Zones (< 2%) except in Ecological Zone 1 where there was a relatively elevated (5.3%) positivity for DENV antibodies, and with the highest overall positivity (8.6%) for the other three flaviviruses combined except ZIKAV. Assessing other works done in East Africa, for example by Abdelhalim, et al. [18] in Sudan**,** the seroprevalence of WNV and DENV IgG antibodies were found to be 64 and 14%, respectively. In another study in Kenya [16], there was 14.4% seropositivity for DENV and 9.5% positivity for WNV. These studies however did not employ the PRNT test to rule out false positivity; hence it is very probable that the real positivity rate would have been lower. The higher DENV positivity rate in Ecological Zone 1 in the current study can in part explain the recurrent dengue fever outbreaks in Gode town (one of the sampling sites of Ecological Zone 1) and indicates the potential for future threats of dengue fever outbreaks in all areas of the Zone.

There are potential limitations of our study. We lacked accurate country-level population data by region, age, and sex and thus were not able to adjust for differences between the sample population and the population of the country. A conservative approach was taken in the classification of vaccination (included all verbal reports of potential vaccination regardless of the timing of the vaccination and whether YF IgM or IgG antibodies were detected). Using historic data on YF seroprevalence to estimate the sample size led to several areas being under sampled and likely limited our power to detect lower levels of YFV seropositivity (e.g., Zone 2). In addition, by equaling distributing the number of participants to be sampled in a Zone between the two points without further adjusting for population size, we again might have limited our power to accurately assess YFV seropositivity. Our estimated participation rate (85%), which was based on other risk assessments [15], used to estimate our sample size was too high for certain locations. This lead us to sample additional households in some of the randomly selected sites effecting the representativeness of our results for some Zones. We assumed that all YFV infections were naturally-acquired and were acquired in the Zone where the participant was sampled. By not accounting for any potential movement, we might have misclassified the YFV risk in the Zones. Given the low seroprevalence of YFV antibodies in the positive Ecological Zones, it may be surmised that generally the risk of a YF epidemic in the country is low. Clearly based on the seroprevalence data, the risk exists but prior to concluding that it is only a minimal risk, other factors need to be considered to make the assessment more realistic.

## Conclusions

The current study showed previous or recent sero prevalence of YFV and related flavi viruses based on IgG antibodies against the viruses. Considering the age of the study participants that had IgG positivity for the viruses considered in this study, it can be concluded that these viruses were circulating very recently with high probability of active circulation. The Sero prevalence data showed low prevalence of YFV and related viruses circulation in Ethiopia. In general, there seems to be a decline in the prevalence of YF antibodies compared to previous studies which reported much higher YF seroprevalence rates, such as after the historic YF outbreaks in Ethiopia half a century ago. The low prevalence of IgG antibodies implies that the proportion of Ethiopian population with protective antibodies against the virus is very low; or in other words, the majority of the population doesn’t have the protection against infection and this puts the population at risk of infection by YFV. There are also other additional factors that indicate that YF could be a problem in Ethiopia and call for the need for YF prevention and control strategy. Firstly, previous outbreaks demonstrated that YF is a problem in Ethiopia, especially in the western and south western parts of the country. Secondly, the fact that there have been recent recurrent vector-borne outbreaks in Ethiopia such as dengue fever outbreaks is an indicator that YF could cause potential outbreak, since the vector for dengue and yellow fever is of the same species. In addition, neighboring countries to Ethiopia such as Sudan and South Sudan have been frequently affected by YF outbreaks and the recent yellow fever risk assessment in Sudan has shown that there is a high yellow fever circulation which necessitated the need to propose a yellow fever mass vaccination.

The current WHO recommendation for YF control is vaccination. Vaccination is the single most important measure for preventing YF. The vaccine is safe and effective and provides immunity within one week in 95% of those vaccinated. YF control is based on prevention of outbreaks, and this can only be achieved if the majority of the population is immunized. A two-pronged strategy is used to achieve this goal. The first strategy is inclusion of the YF vaccine in national childhood routine immunization programs, to be administered at 9 months of age, simultaneously with the first dose of measles vaccine, however reaching the desired level of population immunity takes more than 30 years with infant routine immunization only, even with high coverage. Therefore this strategy needs to be combined with the implementation of mass preventive vaccination campaigns to rapidly increase the population immunity and protect the susceptible older age groups in selected high risk areas. In addition sensitive surveillance for YF needs to be implemented for prompt detection and confirmation of the YFV if it surfaces.

Based on the results of the current serosurvey, we are not recommending a preventive mass vaccination campaign, but considering other YF epidemic risk factors for Ethiopia as well as WHO recommendation for YF control, we propose the introduction of YF vaccine in routine EPI nationwide, to be administered at the age of nine months, together with the first dose of measles vaccine. In addition, robust YF case-based and laboratory supported surveillance should be established nationwide to be able to detect an outbreak and respond before it spreads. It is also important to undertake a well-organized entomological survey nationwide and surveillance to understand the types of arbovirus vectors present and their distribution in the country. This study has also provided the platform for understanding the prevalence and virus activity of other flaviviruses, namely DENV, WNV and ZIKAV, and it is the first study of its kind to determine the prevalence of these other flaviviruses at a national level. Except for Ecological Zone 4, in all the remaining Ecological Zones, there was seropositivity for one or more of the flaviviruses tested in this study. Even though it is important to confirm the current activity and circulation of YFV and the related flavi viruses, this finding warrants that the health sector in Ethiopia needs to consider these viruses as potential agents of mortality, morbidity and outbreaks and the surveillance system need to be vigilant to detect their circulation in communities throughout Ethiopia.

## Abbreviations

DENV: Dengue Virus
ELISA: Enzyme-linked Immunosorbent Assay
EPHI: Ethiopian Public Health Institute
IgG: Immunoglobulin G
IgM: Immunoglobulin M
PRNT: Plaque Reduction Neutralization Test
WHO: World Health Organization
WNV: West Nile Virus
YF: Yellow fever
YFV: Yellow Fever Virus
ZIKAV: Zika Virus
